# Loss of heterozygosity for chromosomal regions 15q14-21.1, 17q21.31, and 13q12.3-13.1 and its relevance for prostate cancer

**DOI:** 10.1007/s12032-015-0691-y

**Published:** 2015-10-03

**Authors:** Maria Nowacka-Zawisza, Ewa Forma, Maciej Walczak, Waldemar Różański, Magdalena Bryś, Wanda M. Krajewska

**Affiliations:** Department of Cytobiochemistry, Faculty of Biology and Environmental Protection, University of Lodz, Pomorska St. 141/143, 90-236 Lodz, Poland; 2nd Department of Urology, Medical University of Lodz, Pabianicka 62, 93-513 Lodz, Poland

**Keywords:** Loss of heterozygosity (LOH), Prostate cancer, RAD51, Molecular markers

## Abstract

Although prostate cancer is one of the most common cancers in men, the genetic defects underlying its pathogenesis remain poorly understood. DNA damage repair mechanisms have been implicated in human cancer. Accumulating evidence indicates that the fidelity of the response to DNA double-strand breaks is critical for maintaining genome integrity. RAD51 is a central player in double-strand break repair via homologous recombination, and its alterations may confer and increase the risk of cancer. RAD51 functioning depends on the indirect or direct interactions with BRCA1 and BRCA2. To evaluate the contribution of RAD51 to sporadic prostate cancer, loss of heterozygosity (LOH) for chromosomal region 15q14-21.1 (*RAD51locus*) was determined and compared to LOH in 17q21.31 (*BRCA1 locus*) and 13q12.3-13.1 (*BRCA2* region). DNA was isolated from prostate biopsies and matched peripheral blood of 50 patients. The regions 15q14-21.1, 17q21.31, and 13q12.3-13.1 were examined using microsatellite markers on chromosome 15 (D15S118, D15S214, D15S1006), chromosome 17 (D17S855, D17S1323), and chromosome 13 (D13S260, D13S290), respectively. The LOH in tumors was analyzed by PCR with fluorescently labeled primers and an ABI PRISM 377 DNA Sequencer. Allele sizing was determined by GeneScan version 3.1.2 and Genotyper version 2.5 software (Applied Biosystems, USA). LOH was identified in 57.5, 23, and 40 % for chromosomal regions 15q14-21.1, 17q21.31, and 13q12.3-13.1, respectively. Twenty-six percent of studied cases manifested LOH for at least one marker in 15q14-21.1 exclusively. A significant correlation was found between LOH for studied region and PSAD (prostate-specific antigen density). The findings suggest that *RAD51* may be considered as a prostate cancer susceptibility gene.

## Introduction

Prostate cancer is the second most common cancer to affect males worldwide. This tumor is one of the most frequently registered cancers among men in Poland, and cases of prostate cancer represent above 14 % of all cancer morbidity and 8 % of cancer mortality among men [1]. As prostate cancer exhibits a diverse spectrum of behavior, management of the disease is controversial [2]. Prostate-specific antigen (PSA) is considered to be the most remarkable prostate tumor marker. However, there is no clear evidence of a relationship between mean PSA levels at screening and the incidence or the rate of cancer detection. Hence, improved biomarkers are needed to enhance prediction of PSA. Various candidates have been proposed to increase the value of PSA as a diagnostic and prognostic marker [3–8]. However, unlike some human malignancies, the etiology of prostate cancer does not appear to be associated with a specific genetic susceptibility but rather with multiple gene loci, each independently conferring a low but cumulative risk. Meta-analysis and genome-wide association studies (GWAS) have mapped the loci for prostate cancer susceptibility to several chromosomes, and although putative candidate genes have also been suggested, their significance for prostate cancer remains unknown [9, 10].

Numerous studies describe an association between mutations in DNA repair genes and neoplastic transformation. Mutations in *BRCA1*, *BRCA2*, *BRIP1*/*FANCCJ*, *CHEK2*, *MMR*, and *NBS1* have been found to confer an increased risk of prostate cancer [11–20]. The response of cells to DNA damage and their ability to maintain genomic stability by DNA repair are critical for preventing cancer initiation and progression. The most dangerous class of genetic material damage is the double-strand break (DSB). The key mode of DNA double-strand break repair includes homologous recombination (HR), which precisely restores the genomic sequence of the broken DNA ends by using sister chromatids as a template for repair [21–23].

Although a plethora of proteins participate in the HR repair of double-strand breaks, a crucial role is played by RAD51, a mammalian homolog of bacterial RecA, an evolutionarily conserved recombinase encoded by the *RAD51* gene located on human chromosome 15q15.1. RAD51 co-localizes with BRCA1 and BRCA2 protein within nuclear foci in mitotic cells. The foci have been observed to contain BRCA1 together with the BRCA1-binding protein BARD1, both before and after DNA damage. RAD51 foci appear during the S-phase and are required to initiate stalled or broken DNA replication forks. RAD51 recombinase forms a direct association with BRCA2, which is essential for normal recombination and genome stability, as the interaction between BRCA2 and RAD51 is fundamental for error-free HR in response to DSBs. While BRCA2 is directly involved in RAD51-mediated repair, BRCA1 acts upstream from this pathway and is thought to be required for the transport of RAD51 from the cytoplasm into the nucleus and sites of DNA damage. BRCA2 contains nuclear localization signals not found in RAD51, supporting the notion that BRCA2 also facilitates RAD51 transport into the nucleus. The direct interactions of RAD51 and BRCA1 have not yet been fully elucidated, despite gene expression profiling and network modeling revealing a complex heterogeneity in the mechanisms of BRCA1 involvement in tumorigenesis [24, 25].

The limited understanding of the genetic elements governing prostate cancer progression demands further studies of its predisposing genes. Since genome instability is a hallmark of a malignant phenotype and a driving force for tumorigenesis, investigations of genes involved in DNA double-strand breaks merit special interest. Although germline mutations and LOH in *BRCA1* and *BRCA2* genes have been detected in mutation carriers, no data currently exist concerning the role of *RAD51* in sporadic prostate cancer [26, 27]. Hence, the aim of the present study is to determine whether loss of heterozygosity (LOH) in *RAD51*, *BRCA1*, and *BRCA2* contributes to the sporadic prostate cancer. Loss of heterozygosity for chromosomal regions 15q14-21.1, 17q21.31, and 13q12.3-13.1 was assessed by seven microsatellite markers. The relationships between the clinicopathological parameters of prostate adenocarcinoma and LOH in studied chromosomal regions were examined.

## Patients and methods

### Patients and tissue samples

Peripheral blood and prostate adenocarcinoma biopsies were collected at the Second Department of Urology, Medical University of Lodz, Poland, between October 2009 and December 2011. All samples were obtained from the peripheral zone of the prostate gland in patients who underwent transrectal ultrasound (TRUS)-guided prostate biopsy. After (TRUS)-guided prostate biopsy, the tissue samples were placed in RNAlater^®^ solution (Qiagen, Inc., Chatsworth, CA, USA) and stored at −70 °C until further analysis. Blood samples from each patient were collected on EDTA and frozen.

All tumor specimens were routinely assessed clinicopathologically for cancer stage and Gleason score by independent pathologists. All other data were taken from patients by diagnostic and epidemiology questionnaires. The following characteristics were recorded: age of patients, the level of prostate-specific antigen PSA, i.e., total PSA (PSAT) and free PSA (PSAF) in the serum of patients measured at the time of diagnosis, and PSA density (PSAD) for tumor tissue and prostate volume. None of the patients had undergone hormonal therapy, radiotherapy, or chemotherapy prior to surgery. Samples were obtained in accordance with ethical and legal requirements. Informed consent was obtained from patients, and the Independent Ethical Committee of the Medical University of Lodz, Poland, approved this study (RNN/59/09/KE).

In total, 50 patients with prostate adenocarcinoma were recruited for this study. The clinical characteristics of the studied material are given in Table 1.

**Table 1:** Clinical characteristics of study subjects

Age (year)	
Range	55–86
Mean ± SD	71.2 ± 8.6
Median	72
<72	24 (48 %)
≥72	26 (52 %)
PSAT (ng/ml)	
Range	4.58–1489
Mean ± SD	104.04 ± 251.28
Median	17.37
≥4–10	15 (30 %)
≥10–20	11 (22 %)
>20	24 (48 %)
PSAF/PSAT	
Range	0.05–0.79
Mean ± SD	0.19 ± 0.13
Median	0.16
<0.16	24 (48 %)
≥0.16	26 (52 %)
PSAD (ng/ml)	
Range	0.08–56.4
Mean ± SD	2.72 ± 8.51
Median	0.29
<0.15	8 (16 %)
≥0.15	42 (84 %)
Prostate volume (ml)	
Range	20.7–191
Mean ± SD	57.72 ± 35.91
Median	47.25
<50	28 (56 %)
≥50	22 (44 %)
Gleason score	
4	2 (4 %)
6	9 (18 %)
7	18 (36 %)
8	11 (22 %)
9	9 (18 %)
10	1 (2 %)
Cancer stage	
T1	12 (24 %)
T2	14 (28 %)
T3	12 (24 %)
T4	12 (24 %)

### DNA isolation

The DNA from prostate biopsies was isolated using TRI Reagent^®^ (Sigma-Aldrich, USA). The DNA from peripheral blood of patients with prostate adenocarcinoma was isolated using AxyPrep™ Blood Genomic DNA Miniprep Kit (Axygen, USA) as a reference. DNA purity and quantity were estimated by UV spectroscopy (Eppendorf BioPhotometer TM Plus, Eppendorf, Germany). DNA was identified by a ratio of 260/280 nm ranged 1.8–2.0.

### PCR conditions and primers

Genetic alterations for chromosomal regions: 15q14-21.1, 17q21.31, and 13q12.3-13.1 were analyzed for seven microsatellite markers. Information about the microsatellite markers and the sequences for all the primers for LOH analysis was obtained from the National Center for Biotechnology Information—NCBI (www.ncbi.nlm.nih.gov). The primers were synthesized and labeled fluorescently by Applied Biosystems (USA). Polymerase chain reaction (PCR) was carried out in a 10 μl reaction volume containing 50 ng genomic DNA, 1× Solis Biodyne Buffer B1, 3U HOT FIREPol^®^ DNA polymerase, 200 μl GeneAmpdNTP Mix, 2 mM MgCl_2_, and 10 pmol/μl primers, of which one was end-labeled with 6-FAM or TET phosphoramidite dye. PCR reagents were obtained from Solis Biodyne (Estonia) and Applied Biosystems (USA). Each microsatellite marker was amplified at its own specific annealing temperature to optimize the PCR reaction. Profile times and temperatures were as follows: 12 min at 95 °C; 30 amplification cycles comprising denaturation for 15 s at 95 °C, primer annealing for 30 s at 55 °C (for microsatellite markers: D15S118, D15S214, D15S1006, D17S1323, D13S260) or 51 °C (for microsatellite markers D17S855, D13S290), elongation for 30 s at 72 °C; 10 min for 72 °C. Amplification was performed in a GeneAmp 2400 thermal cycler (PerkinElmer, USA). Localization of the analyzed microsatellite markers and primer sequences are presented in Table 2.

**Table 2:** Characteristics of the analyzed microsatellite markers and PCR reaction (http://www.ncbi.nlm.nih.gov)

Microsatellite marker	Chromosomal region (gene)	Position	Primer sequence	Dye	Product sizes (bp)
D15S118	15q14	12 996 879	(+) TCAAAGACCCATATCAACCA	6-FAM	218–232
			(–) GTGCTGAAAAGCGACACTTA		
D15S214	15q15.1 (*RAD51*)	17 166 170	(+) GGAGGGCACTTCCTGAG	TET	260–274
			(–) GCCTGGCATCACGACT		
D15S1006	15q21.1	24 439 646	(+) AGGGAATACTTCAAAACTC	6-FAM	212–224
			(–) CCACTTGGCTATGGTGAAT		
D17S855	17q21.31 (*BRCA1*)	37 861 601	(+) GGATGGCCTTTTAGAAAGTGG	6-FAM	142–156
			(–) ACACAGACTTGTCCTACTGCC		
D17S1323		37 894 900	(+) TAGGAGATGGATTATTGGTG	TET	153–161
			(–) AAGCAACTTTGCAATGAGTG		
D13S260	13q12-13 (*BRCA2*)	13 503 800	(+) AGATATTGTCTCCGTTCCATGA	6-FAM	155–171
			(–) CCCAGATATAAGGACCTGGCTA		
D13S290		12 495 878	(+) CCTTAGGCCCCATAATCT	TET	176–190
			(–) CAAATTCCTCAATTGCAAAAT		

### LOH analysis

**Fig. 1:**
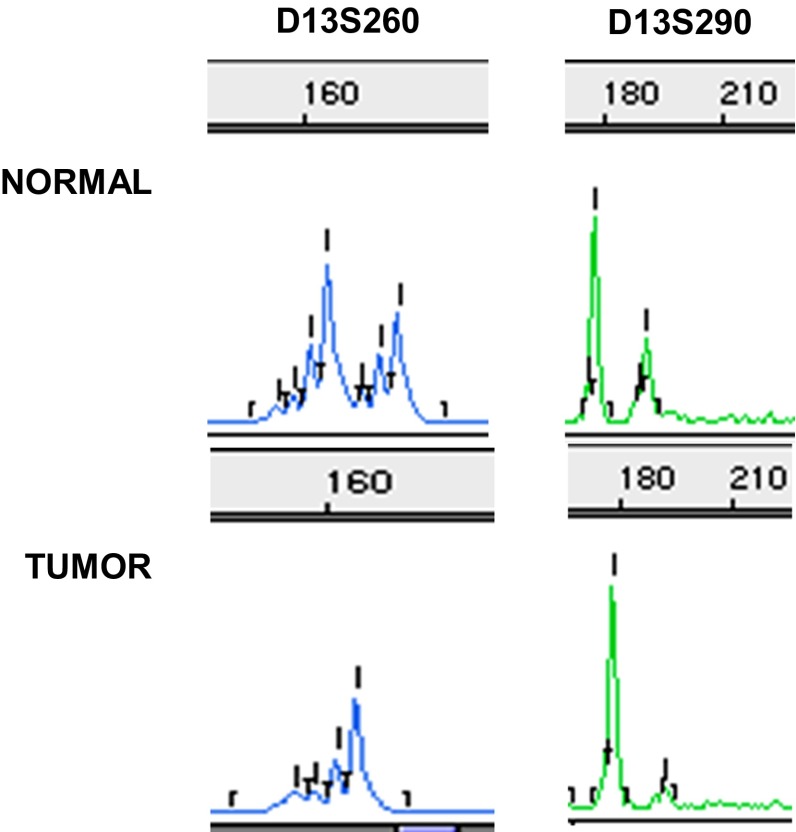
Example of LOH analysis for 13q12-13 region in prostate cancer

PCR products were electrophoresed on polyacrylamide gel (5 % Long Ranger) containing 6 M urea and 1 × TBE (10 × TBE: Tris borate, EDTA, pH 8.0). After PCR, the samples were mixed with stop solution containing deionized formamide, GeneScan-350 TAMRA Size Standard, and loading buffer (blue dextran, EDTA), denatured, and chilled on ice. Three microliters of each sample mixture was applied in each well and run on an ABI PRISM 377 DNA Sequencer (Applied Biosystems, USA). The data were collected automatically. Allele sizing was determined by GeneScan v. 3.1.2 and Genotyper v. 2.5 software (Applied Biosystems, USA). Amplification of microsatellite markers will yield one or two major allele peaks, depending upon whether the individual is homozygous (non-informative cases) or heterozygous (informative cases) for that marker. LOH was defined as loss or ≥50 % reduction in either allele in cancer compared with peripheral blood derived from the same patient. Allele ratios were calculated for informative cases as described by Cawkwell et al. [28] according to the formula T1 × N2/T2 × N1, where T1 and N1 are the values of the shorter length allele and T2 and N2 of the longer length allele for the tumor (T) and normal (N) sample, respectively. A representative sample with LOH in D15S118 and D15S214 microsatellite markers for 15q14-21.1 region in prostate cancer as compared to normal tissue is presented in Fig. 1. For each prostate cancer sample, fractional allele loss (FAL) index was also calculated reflecting the ratio of total number of chromosomal *loci* with LOH to the total number of informative loci examined.

### Statistical analysis

The statistical significance of LOH for at least one microsatellite marker identified concomitantly in three studied regions was tested using Spearman rank correlation test. Statistical significance of the relationship between the presence of LOH and clinicopathological parameters was performed using Fisher’s exact test. *P* < 0.05 was considered statistically significant. For associations between clinical variables of patients (age at diagnosis, total PSA (PSAT), total/free PSA value (PSAF/PSAT), PSA density (PSAD), prostate volume), histopathological parameters (according to TNM classification and Gleason score), and FAL index values, a Mann–Whitney *U* test or Kruskal–Wallis test was performed. The correlation between presence of LOH and the level of PSA and patients age was examined using linear regression. The statistical analysis was carried out using the Statistica for Windows, v. 5.

## Results

The loss of heterozygosity (LOH) was evaluated using the microsatellite markers D15S118, D15S214, and D15S1006 for chromosomal region 15q14-21.1, D17S855, and D17S1323 for chromosomal region 17q21.31, and D13S260 and D13S290 for chromosomal region 13q12.3-13.1. The LOH studies rely on the detection of the loss of a single copy of the two alleles or ≥50 % reduction in either allele. Those with detectable heterozygous alleles were defined as informative cases. Frequency of LOH for studied microsatellite markers in prostate cancer is presented in Table 3.

**Table 3:** Frequency of LOH in prostate cancer for studied microsatellite markers

Sample no.	D15S118	D15S214	D15S1006	D17S855	D17S1323	D13S260	D13S290	FAL index
1	ni	ni	ni	NG	NG	NG	ni	0
2	NG	NG	NG	ni	NG	ni	NG	0
3	ni	ni	ni	NG	ni	LOH	LOH	0.667
4	NG	LOH	NG	ni	ni	ni	ni	0.333
5	ni	LOH	NG	NG	ni	NG	NG	0.5
6	ni	ni	NG	ni	ni	NG	LOH	0.5
7	ni	LOH	ni	ni	NG	ni	ni	0
8	NG	LOH	LOH	ni	NG	ni	ni	0.667
9	ni	ni	NG	ni	ni	NG	ni	0
10	NG	ni	ni	ni	ni	ni	NG	0.5
11	NG	ni	ni	ni	ni	NG	ni	0.667
12	ni	LOH	ni	ni	NG	LOH	ni	1
13	NG	NG	ni	NG	ni	LOH	ni	0.4
14	ni	ni	ni	NG	ni	ni	ni	0.6
15	ni	ni	ni	NG	ni	ni	NG	0.333
16	NG	ni	ni	ni	ni	ni	ni	0.2
17	NG	ni	ni	ni	ni	LOH	ni	0
18	ni	ni	LOH	ni	ni	LOH	ni	1
19	LOH	LOH	ni	ni	NG	ni	ni	0
20	ni	ni	ni	NG	ni	NG	ni	0.5
21	ni	NG	ni	ni	ni	ni	LOH	0
22	ni	ni	ni	ni	ni	NG	NG	1
23	NG	LOH	ni	ni	NG	NG	ni	0
24	NG	LOH	ni	ni	ni	LOH	ni	0
25	LOH	LOH	ni	LOH	ni	LOH	ni	0.75
26	NG	LOH	ni	ni	ni	ni	NG	0
27	NG	ni	ni	LOH	NG	ni	ni	0
28	LOH	NG	ni	LOH	ni	ni	ni	0
29	ni	LOH	ni	ni	ni	ni	ni	0.5
30	ni	ni	ni	NG	ni	ni	NG	0.5
31	LOH	ni	ni	NG	ni	LOH	LOH	0.5
32	LOH	ni	LOH	NG	ni	ni	ni	0
33	LOH	NG	ni	ni	ni	ni	ni	0.667
34	LOH	LOH	ni	NG	ni	NG	NG	1
35	LOH	NG	ni	LOH	ni	NG	LOH	0.75
36	ni	ni	ni	ni	ni	NG	NG	0.25
37	NG	ni	ni	NG	NG	ni	ni	0
38	NG	ni	ni	ni	NG	NG	LOH	0
39	ni	ni	ni	ni	LOH	NG	ni	0
40	ni	LOH	ni	ni	NG	ni	ni	0.25
41	NG	LOH	NG	LOH	NG	NG	ni	0
42	ni	NG	ni	ni	ni	NG	NG	0.5
43	ni	NG	ni	NG	ni	NG	NG	0.25
44	ni	LOH	ni	ni	ni	ni	ni	0.333
45	ni	NG	LOH	ni	ni	NG	LOH	0.333
46	ni	ni	NG	ni	ni	ni	ni	0.667
47	ni	ni	ni	ni	ni	NG	NG	0
48	LOH	ni	ni	NG	LOH	Ni	LOH	0.667
49	ni	NG	NG	ni	NG	NG	ni	0
50	ni	ni	NG	NG	NG	ni	NG	0.333

*LOH* loss of heterozygosity, *NG* heterozygous without LOH, *ni* non-informative cases (homozygous)

As shown in Table 4, genomic deletion detected by allelic loss varied according to the region tested. In the case of chromosomal region 15q14-21.1, loss of heterozygosity was observed in 57.5 %, i.e., in 23 out of 40 heterozygous patients. In the region 15q14-21.1, the highest incidence of LOH, corresponding to 60 % of informative cases, was found for microsatellite marker D15S214 located at *RAD51 locus*. In the 17q21.31 region, LOH occurred in 23 %, i.e., in 7 out of 30 heterozygous patients, and in the 13q12.3-13.1 region in 40 %, i.e., in 14 out of 35 heterozygous patients. Twenty-six percent of the studied prostate cancer cases displayed LOH for at least one microsatellite marker at 15q14-21.1 region, without LOH in the 17q21.31 or 13q12.3-13.1 regions. The LOH for at least one microsatellite marker in the 17q21.31 and 13q12.3-13.1 regions, without LOH in 15q14-21.1 region, was estimated as only 6 and 19 %, respectively.

**Table 4:** Incidence of LOH at 15q14-21.1, 17q21.31, and 13q12-13 chromosomal regions in prostate cancer

Chromosomal region	Number of informative case/studied cases (%)	Number of tumor with LOH/informative cases (%)
15q14-21.1	40/50 (80 %)	23/40 (57.5 %)
17q21.31	30/50 (60 %)	7/30 (23 %)
13q12.3-13.1	35/50 (70 %)	14/35 (40 %)

The association of the LOH in studied chromosomal regions with clinicopathological characteristics of patients was analyzed using Fisher’s exact test. A significant correlation was found between LOH in chromosomal regions 15q14-21.1 and 13q12.3-13.1 and PSA density (PSAD) (Table 5).

**Table 5:** Relationship between LOH at 15q14-21.1, 17q21.31, and 13q12-13 regions and the clinicopathological characteristics of prostate cancer

Clinical characteristics	Chromosomal region											
	15q14-21.1				17q21.31				13q12.3-13.1			
	*I*	*N*	LOH	*P*	*I*	*N*	LOH	*P*	*I*	*N*	LOH	*P*
Tumor cases	40	17	23		30	23	7		35	21	14	
Age												
<72	18	7	11	0.755	16	11	5	0.399	21	13	8	>0.999
≥72	22	10	12		14	12	2		14	8	6	
PSAT (ng/ml)												
≥4–10	14	6	8	0.088	8	7	1	0.284	11	5	6	0.383
>10–20	6	5	1		5	5	0		9	7	2	
>20	20	6	14		17	11	6		15	9	6	
PSAF/PSAT												
<0.16	18	7	11	0.755	15	13	2	0.390	17	11	6	0.733
≥0.16	22	10	12		15	10	5		18	10	8	
PSAD (ng/ml)												
<0.15	8	2	6	0.049	7	4	3	0.306	7	1	6	0.001
≥0.15	32	15	17		23	19	4		28	20	8	
Prostate volume (ml)												
<50	22	11	11	0.348	14	11	3	>0.999	21	16	5	0.033
≥50	18	6	12		16	12	4		14	5	9	
Gleason score												
<7	15	7	8	0.749	6	4	2	0.603	10	6	4	>0.999
≥7	25	10	15		24	19	5		25	15	10	
Cancer stage												
T1–T2	21	8	13	0.749	13	11	2	0.427	19	11	8	>0.999
T3–T4	19	9	10		17	12	5		16	10	6	

*P* < 0.05 indicates significant association, *I* Informative cases, *NG* heterozygous without LOH (negative cases)

**Fig. 2:**
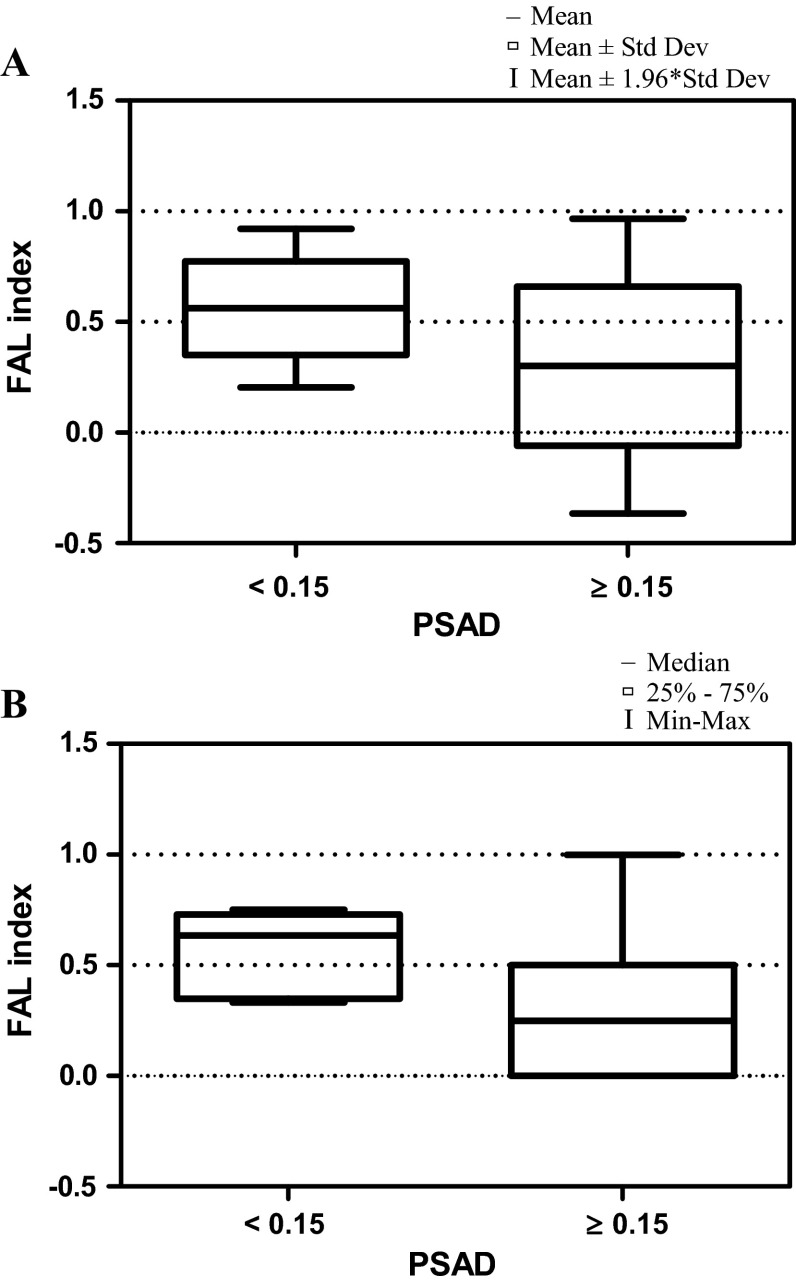
*Box*-and-*whisker plot*, representing the **a** mean and **b** median FAL index values for the PSAD in prostate cancer

Mann–Whitney *U* test revealed statistically significant differences between FAL index levels and PSA density (Fig. 2). There was no statistically significant correlation between FAL index levels and patients’ age, free to total PSA value, prostate volume, Gleason score, and TNM classification (Mann–Whitney U test) as well as total PSA level (Kruskal–Wallis test). Neither correlation between age of patients with LOH and PSA level, free to total PSA value nor PSA density was identified (data not shown).

## Discussion

Although the contribution of the homologous recombination mediators *BRCA1* and *BRCA2* to prostate cancer has been previously investigated, this is the first study to address the importance of *RAD51* genetics in the occurrence of sporadic prostate cancer [26, 29]. RAD51 is a relatively small and rigid protein playing a basic role in the high-fidelity DNA repair mechanism of homologous recombination via homology search and DNA strand exchange. RAD51 appears vital for cell survival, as its depletion results in embryonic lethality. It has been highly conserved throughout evolution, and until now, no single mutation has been detected in the coding region of *RAD51* in any type of cancer. However, a strong correlation has been identified between its expression level and both cancer development and progression. It is suggested that overexpression of RAD51 suppresses recombination defects [24, 30–32].

The progression of prostate cancer as in other malignancies is characterized by increased genetic and epigenetic aberrations. Of particular interest are germline polymorphisms and allelic imbalances, which may affect tumor suppressor genes and oncogenes [8, 33–35].

The *BRCA1* and *BRCA2* genes, whose best known function is concerned with the DNA damage response, have been reported to contribute to prostate cancer, although to different extents. The Breast Cancer Linkage Consortium report increased prostate cancer risk of *BRCA1* mutation carriers below the age of 65, with a relative risk of 1.82, but no increase in those aged over 65. For *BRCA2* mutation carriers, the relative risk of developing prostate cancer was estimated as 4.65, rising to 7.33 for men younger than 65 years [36–39]. Uchida et al. [40] identified LOH in the *BRCA1* gene in primary prostate cancer using seven highly polymorphic tandem repeat markers on chromosome 17q21, in addition to an analysis of the whole coding region of the *BRCA1* gene. Four of the 24 prostate cancer specimens revealed the presence of LOH at one or more loci, all of which were found to be at stage D with poor histological differentiation. One of the 24 cases showed a germline mutation of the *BRCA1* gene. Willems et al. [41] observed LOH at *BRCA2* in 10 of 14 tumors from *BRCA2* mutation carriers (71 %), but no LOH in *BRCA1* in four tumors from *BRCA1* mutation carriers. Assuming that LOH occurs only because the cancer is caused by the germline mutation, carriers of BRCA2 mutations are at 3.5-fold increased risk of prostate cancer. Similarly Edwards et al. [37] identified LOH in the majority of tumors of *BRCA2* mutation carriers. On the other hand, Willems-Jones et al. [42] note that high-grade prostatic intraepithelial neoplasia, believed to be a precursor to prostate adenocarcinoma in some cases, does not display LOH at the mutation locus in *BRCA2* mutation carriers with aggressive prostate cancer.

The present study reveals in prostate cancer the presence of LOH in 57.5, 23, and 40 % for chromosomal regions 15q14-21.1, 17q21.31, and 13q12.3-13.1, respectively. In the region 15q14-21.1, the highest incidence of LOH corresponding to 60 % was found for microsatellite marker D15S214 located at *RAD51 locus*. Hence, chromosomal region 15q14-21.1 was found to display a higher incidence of LOH than *BRCA loci*, especially *BRCA2*, which is considered a prostate tumor suppressor. In prostate cancer, 26 % of cases manifested LOH at 15q14-21.1 chromosomal region exclusively, without LOH in the 17q21.31 or 13q12.3-13.1 regions. Interestingly, LOH in 15q14-21.1 chromosomal region appeared to be related to PSA density.

## Conclusions

The high frequency of allelic losses at the RAD51 locus indicates the important role played by this gene in prostate cancer and sheds light on the novel perspective of genetic changes associated with its development. RAD51 displayed a higher incidence of LOH than BRCA2, which is considered a prostate tumor suppressor. A better understanding of the molecular basis of prostate cancer may permit a more accurate assessment of this disease.
